# T Cell–Macrophage Interactions and Granuloma Formation in Vasculitis

**DOI:** 10.3389/fimmu.2014.00432

**Published:** 2014-09-12

**Authors:** Marc Hilhorst, Tsuyoshi Shirai, Gerald Berry, Jörg J. Goronzy, Cornelia M. Weyand

**Affiliations:** ^1^Division of Immunology and Rheumatology, Department of Medicine, Stanford University, Stanford, CA, USA; ^2^Department of Pathology, Stanford University, Stanford, CA, USA

**Keywords:** macrophage, dendritic cell, T cell, granuloma, vasculitis

## Abstract

Granuloma formation, bringing into close proximity highly activated macrophages and T cells, is a typical event in inflammatory blood vessel diseases, and is noted in the name of several of the vasculitides. It is not known whether specific properties of the microenvironment in the blood vessel wall or the immediate surroundings of blood vessels contribute to granuloma formation and, in some cases, generation of multinucleated giant cells. Granulomas provide a specialized niche to optimize macrophage–T cell interactions, strongly activating both cell types. This is mirrored by the intensity of the systemic inflammation encountered in patients with vasculitis, often presenting with malaise, weight loss, fever, and strongly upregulated acute phase responses. As a sophisticated and highly organized structure, granulomas can serve as an ideal site to induce differentiation and maturation of T cells. The granulomas possibly seed aberrant Th1 and Th17 cells into the circulation, which are known to be the main pathogenic cells in vasculitis. Through the induction of memory T cells, aberrant innate immune responses can imprint the host immune system for decades to come and promote chronicity of the disease process. Improved understanding of T cell–macrophage interactions will redefine pathogenic models in the vasculitides and provide new avenues for immunomodulatory therapy.

## Introduction

Protecting the host from infection and maintaining tissue integrity relies on two highly complex and evolutionary distinguished systems, the innate and adaptive immune system. The two arms of the immune system have developed a sophisticated and efficient crosstalk to defend the host. Monocytes that come into contact with penetrating pathogens differentiate into specialized antigen-presenting cells (APC), such as macrophages and dendritic cells (DC) (1). After phagocytosis and degradation of the pathogen, proteins are presented to specialized cells of the adaptive immune system, specifically T cells. Interactions between macrophages and T cells are critical in the communication between innate and adaptive immunity. Errors in this interaction may result in immunodeficiency, failure to destroy invading pathogens, or damage to host–tissues in the form of autoimmunity. Although the principal function of macrophages was recognized a long time ago (2), the precise mechanisms of macrophage physiology are only now beginning to be unraveled.

Chronic (aberrant) macrophage–T cell interaction leads to the formation of organized lymphoid organ structures, such as granulomas. Granulomas are typically formed during infection, especially when the host has difficulties to eliminate the infectious organism. Classic examples are granulomas induced by *Mycobacterium tuberculosis* infection, often considered a mechanism to contain the infectious organism (3). Granuloma formation is equally important in non-infectious disease states, such as inflammatory blood vessel disease. In giant-cell arteritis (GCA; formerly known as temporal arteritis), granulomas are an almost obligatory part of the disease process. In granulomatosis with polyangiitis (GPA; formerly known as Wegener’s granulomatosis), granuloma formation is captured in the disease name. An important issue in granulomatous diseases is whether the highly activated macrophages building the granulomatous structures have primarily a protective function or whether they are key drivers of tissue damage and disease propagation (4).

In the current review, we compare and contrast the interaction of macrophages and/or DC with T cells in the context of granuloma formation and vasculitis and focus on GCA and GPA as quintessential model systems of how the interface between innate and adaptive immunity contributes to disease pathogenesis.

## Macrophages and Dendritic Cells Influence T Cells

Monocytes relocate to inflammatory lesions upon sensing a chemokine gradient (5) and can differentiate into distinct types of APC on site. A discussion of the similarities and differences between DC and macrophages is beyond the scope of this review (6). Macrophage subtypes form two main groups: M1 or classically activated macrophages (CAM) and M2 or alternatively activated macrophages (AAM). M1 generally specialize in amplifying inflammatory reactions and produce high levels of TNFα, IL-6, and IL-1β. In contrast, M2 are primarily active in tissue repair and their product profile includes IL-10, TGF-β, and growth factors. An active TGF-β pathway results in suppression of inducible nitric oxide synthase (iNOS) expression and NO secretion in macrophages, deviating the cells away from M1 differentiation (7). M1 have been described as “fighting” or “soldier” cells and M2 as “fixing” or “repair” cells (8, 9). The M2 or AAM subtype is not as well defined and much debated (4). It is plausible that monocytes can differentiate into macrophage subtypes positioned somewhere on the M1–M2 or CAM–AAM continuum and are endowed with varying adaptability and plasticity (8, 10).

## Antigen Recognition and Presentation

Macrophages recognize pathogens through so-called pathogen associated molecular patterns, which are detected through Toll-like receptors (TLR) (11, 12), thus distinguishing between self and non-self. As critical recognition structures, TLR enable the build-up of a defensive immune response, they also participate in shaping immune responses underlying autoimmunity (13, 14). To orchestrate tissue cleanup and repair, macrophages must be able to recognize and remove modified host proteins and lipids, e.g., oxidized proteins and lipids. Such products are often described as danger-associated molecular patterns and require competent TLR as recognition structures (15). Oxidation of host proteins, lipids, and nucleic acids results from the action of reactive oxygen species (ROS), often derived from activated macrophages themselves. The latter process has been implicated in the development and propagation of atherosclerosis (16). Importantly, T cells also express TLR, but it is currently unknown what the precise role of these receptors is in modulating T cell function (14, 17).

## Macrophage-Induced Polarization of T Cell Differentiation

Macrophages are principal regulators of immunity by processing and presenting antigens to T cells (18), which are charged with distinguishing self from non-self (19). Antigen recognition by T cells involves the highly polymorphic major histocompatibility complex (MHC) molecules classes I and II (20, 21), which selectively bind antigen peptides and present them on the surface of APC. While T cell receptors bind to HLA–peptide complexes, costimulatory molecules such as CD28 are co-ligated, a mechanism that is mandatory for a more powerful induction of T cell activation (22–24). After entering the T cell activation cascade, T cells differentiate into distinct functional lineages. Some of them become effector cells; others specialize as memory T cells and position themselves in lymphoid storage sites (25). The fate of individual T cells is ultimately shaped by the microenvironment, composed of cytokines, chemokines, and tissue-specific signals (26). The exact mechanism by which macrophages induce activation, proliferation, and differentiation of T cells is incompletely understood (27). Antigen dose, the type of APC and the contact between APC and T cell are all important variables (28). It has been proposed that DC are more powerful partners of naïve T cells and preferentially interact with T cells in organized lymphoid tissue. Conversely, macrophages function as APC for naïve and memory T cells, encountering them in peripheral tissue lesions (29). DC are thought to skew CD4^+^ cells toward Th1 differentiation in an IL-12 dependent manner (30). Other studies have demonstrated a similar effect of macrophages on CD4^+^ cells (31). DC that have been activated by inflammatory mediators can stimulate Th1 proliferation *in vitro*, but these same DC could not do so *in vivo*, possibly due to lacking pathogen contact, resulting in diminished IL-12p40 production (32).

Importantly, one study found inflammatory DC to be more potent inducers of Th17 cells when compared to inflammatory macrophages. The authors concluded that this difference was reflective of differential IL-23 production, which was observed in inflammatory DC but not in macrophages (33). In contrast, other DC subsets have been implicated in inducing regulatory T cells by virtue of producing TGF-β or expressing PD-L1 (34). Suppression of T cell function and proliferation may also result from the local action of IL-10 and TGF-β, typically secreted by M2 macrophages. Gut-residing macrophages have been implicated in inducing regulatory T cells, whereas DC were found to induce Th17 cells by secreting IL-6 combined with TGF-β and possibly IL-23 (35).

## Macrophage-Induced Inhibition of T Cells

Generally, macrophages inhibit the proliferation of T cells via cell–cell contact. Abundantly studied inhibitory mechanisms in T cells are the programed death (PD1) and cytotoxic T lymphocyte antigen (CTLA) pathways. PD1 and CTLA-4 are found on T cells and mediate inhibitory signals when engaged by their respective ligands expressed on the surface of interacting macrophages (36). Malfunctioning of these inhibitory signals, e.g., due to polymorphisms, increases susceptibility for autoimmunity (37–39). Both DC and macrophages express membrane-integrated ligands for PD-1 and CTLA-4 (24). Blockade of PD-L1 on DC is a powerful mechanism to enhance T cell proliferation and cytokine release (34, 40). Besides polymorphisms in *PD-1* and *CTLA-4*, a series of gene polymorphisms, including genes relevant for cytoplasmic signaling pathways, have been associated with susceptibility for autoimmunity (41, 42). As minor variations in threshold settings of cytoplasmic signaling cascades have the potential to bias immune interactions profoundly, it is likely that they impact macrophage–T cell interactions both by accelerating as well as downregulating immune responses.

Another concept has been that resting macrophages preferentially dampen immune responses. Accordingly, resting macrophages have been reported to induce allogeneic T cell anergy, partly by enhancing regulatory T cells. In one study, T cells proliferated when co-cultured with immature DC, became anergic when in a second co-culture with macrophages and proliferated when co-cultured with mature DC in a third co-culture, finally producing IL-2 and IFN-γ. Immature and mature DC both expressed high levels of MHC class II (HLA-DR), but macrophages did not. The costimulatory molecules CD80 and CD86 were present at higher density on mature DC than on immature DC or on macrophages (43). These studies support the notion that APC functions of macrophages and DC are fundamentally distinct.

In some infectious settings, specifically in filarial and yeast infections, the pathogen undermines protective immune responses by inducing macrophages and DC that are able to suppress T cell activation (44, 45). This mechanism is dependent on the production of TGF-β and/or IL-10 in combination with a lack of IL-6. Also, co-culturing allogeneic naïve CD4^+^ T cells with immature DC has been reported to lead to the expansion of IL-10 producing T cells, whereas mature DC promote the proliferation of Th1 cells (46).

Under hypoxic conditions, macrophages were found to suppress the proliferation of T cells via hypoxia-inducible factor 1α (HIF-1α). More HIF-1α knockout macrophages were necessary to suppress T cells as compared to wild-type macrophages (47). HIF-1α may enhance the production of nitric oxide species, which directly suppress T cell proliferation. In contrast, under hypoxic conditions, which enrich the environment for macrophage-derived oxidative species, T cells preferentially differentiated into Th17 cells. HIF-1α-dependent proteosomal degradation of the transcription factor Foxp3 and enhancement of IL-17 expression via RORγt and Stat3 have been proposed as the underlying mechanism (48). The HIF-1α dependent processes have been shown to play an important role in rheumatoid arthritis and may be of importance in other autoimmune diseases (49).

## T Cells Regulate the Maturation of Macrophages and Dendritic Cells

The differentiation of monocytes into macrophages is a rapid process controlled by cytokines in the environment and cell–cell interactions (10). Thus, neighboring cells can effectively regulate the induction, functional differentiation, and the survival of macrophages (1, 50). It is believed that the polarization of monocytes into M1 or M2 can occur in the absence of T cells (51). However, generally it is assumed that Th1 cells skew monocytes toward M1 whereas Th2 cells skew monocytes toward M2 (4). The lineage commitment of M1 and M2 cells has been associated with the induction of distinct arginine metabolical pathways (52). How T cells regulate this process, however, is not understood.

IL-17 was shown to induce macrophages to produce high levels of IL-6, IL-1β, and TNFα, as well as lower levels of IL-10, IL-12, and PGE_2_ (53), suggesting that Th17 cells bias monocytes toward an M1-like phenotype. Another study demonstrated that pretreatment of monocytes with IFN-γ (in addition to IL-10 and glucocorticoids) prevented the differentiation of monocytes into M2c cells. Instead, IFN-γ-treated monocytes had a higher expression of Fas and were apoptosis susceptible. Interestingly, monocytes treated with IL-17 (in addition to IL-10 and glucocorticoids) differentiated into M2c and had enhanced phagocytic capacity (54). In essence, Th17 cells may regulate phagocytic effector functions. *In vivo*, however, the source of IL-17 can be heterogeneous since neutrophils, DC, and macrophages are all capable to produce IL-17, although in low amounts (55, 56).

In mice, CD4^+^CD25^+^ regulatory T cells exert a potent suppressive effect on splenic APC, which cannot be overcome by preactivation (57). In humans, CD4^+^CD25^+^Foxp3^+^ regulatory T cells were found to direct monocytes toward M2; characterized by high surface expression of CD206 and CD163 but low levels of HLA-DR (58). Also, human CD4^+^CD25^+^ T cells decrease the production of TNFα and IL-6 and increase the production of IL-10 in co-cultured monocytes (59). Murine CD4^+^CD25^−^Foxp3^−^cells can temper the production of proinflammatory cytokines in macrophages via close cell–cell contact. Biologic relevance of this mechanism is suggested by the observation that in the absence of CD4^+^ T cells, innate immune responses are so vigorous that they cause a cytokine storm and death (60). A more recent murine study has demonstrated that both memory and effector CD4^+^ T cells decrease IL-1β production in bone marrow-derived macrophages without affecting TNFα or IL-6 production, possibly by selective inhibition of the inflammasomes NLRP3 and NLRP1 (61).

In summary, selected macrophages and DC can shape T cell differentiation through the secretion of IL-1β, IL-6, and TNFα, whereas other macrophages and DC can suppress T cells via cell–cell contact and the secretion of IL-10 and TGF-β (Table 1). Vice versa, Th1 and Th17 activate macrophages via IFN-γ and IL-17, respectively. Regulatory T cells can suppress the activity of macrophages by secreting IL-10, thus driving them toward the M2 phenotype (Table 2).

**Table 1:** **Summary of macrophage products in relation to possible effects on T cells**.

	Producer	Giant-cell arteritis	Granulomatosis with polyangiitis	Effect
IL-1β	M1	++	+++	Proinflammatory
IL-6	M1	++	+	Proinflammatory
IL-8	M1	+/−	+	Proinflammatory
IL-18	M1/M2	unknown	+	Neutrophil attractant and primer
TNFα	M1	+	+	Proinflammatory; granuloma formation
IL-23	M1	+	+	Th17 sustaining
CCL2	M1	+	+	Monocyte attractant
PGE_2_	M1	unknown	+	Phagocytosis
IL-15	M1/M2	unknown	+	T cell and NK-cell activation; vitamin D pathway
Vitamin D	M1/M2	unknown	+	Anti-inflammatory
Osteopontin	M2	unknown	++	Monocyte and neutrophil chemoattractant
VEGF	M2	++	+	Neoangiogenesis
PDGF	M2	++	+	Tissue remodeling and repair
TGF-β	M2	+	++	Anti-inflammatory; Th17 inducing
IL-10	M2	+	+	Anti-inflammatory; Treg inducing

*Macrophage products involved in vasculitis*.

**Table 2:** **Summary of T cell products in relation to possible effects on macrophages**.

	Producer	Giant-cell arteritis	Granulomatosis with polyangiitis	Effect
IL-17	Th17	++	++	Proinflammatory; M1 supporting
IL-21	Tfh; Th17	++	+	Proinflammatory
IL-22	Th22	unknown	unknown	Proinflammatory
GM-CSF	Th17	+	unknown	M1 supporting
IFN-γ	Th1	++	++	Proinflammatory; M1 supporting; drives multinucleation
IL-27	Th1	+	unknown	Proinflammatory
IL-32	Th1	+	+	Proinflammatory
IL-10	Treg	+/−	+	Anti-inflammatory; M2 supporting

*T cell products involved in vasculitis and granulomatous microenvironments*.

## Granuloma Formation

**Figure 1:**
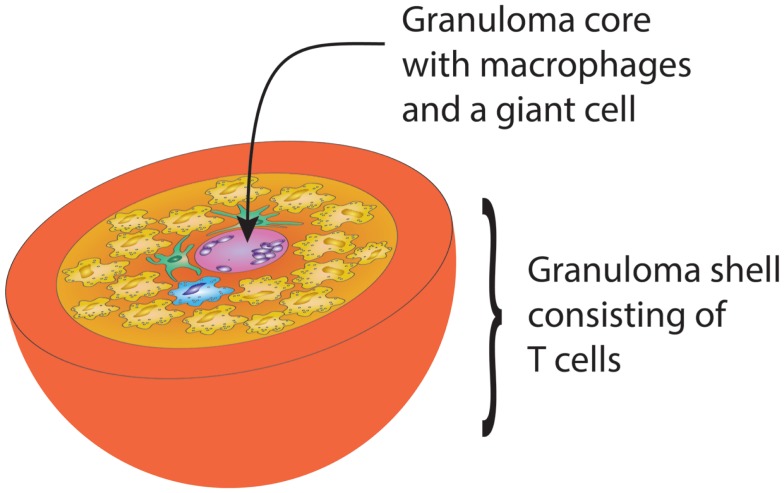
**Schematic drawing of the gross architecture of a granuloma with macrophages, dendritic cells, and multinucleate giant-cells forming the core of the sphere, surrounded by a shell of lymphocytes**. By clustering innate and adaptive immune cells, the granuloma has great potential to induce and perpetuate immune responses, but is equally powerful in causing damage to surrounding tissues.

Designed to protect the host from infection and cancer, the adaptive immune system displays complex microarchitectures to optimize immune responses. One of these lymphoid microstructures has been named granuloma and typically consists of a sphere of highly activated macrophages, surrounded by a shell, i.e., a peripheral layer of T lymphocytes (Figure 1) (62). The current paradigm holds that antigens that are difficult-to-eliminate are prone to elicit granuloma formation. Persistent particulate substances, such as silica, beryllium, or zirconium, but also suture material, are often considered as typical triggers of non-infectious granulomas (63). Difficult-to-eliminate antigens eventually induce the palisading of monocytes/macrophages, which depend on activating signals from other cell populations to form the sophisticated structure of a granuloma. Lymphocytes, especially CD4^+^ T cells, and DC consistently participate in granulomatous infiltrates, but neutrophils, eosinophils, and B cells have also been described (64). Cells are attracted to granulomas by chemokines, cytokines such as interleukins and complement breakdown products. Over time granulomas mature, resulting in the formation of multinucleated giant-cells and epithelioid cells. The precise composition of granulomas may differ according to the inciting agent or pathogen, but the overall architecture of granulomas is usually maintained.

Much of the knowledge on granulomas originates from studying the model system of *M. tuberculosis* infection (3), which remains one of the most prevalent and lethal infectious diseases on the planet (65). It is believed that tuberculous granuloma formation reflects a strategy of the immune system to encapsulate the infection and prevent spreading throughout the body. Tuberculous granulomas have been a rich source of information on the bi-directional interaction between macrophages and T cells (66, 67). One of the hallmark events on the side of macrophages and DC are cell–cell fusions, resulting in multinucleated giant cells. Why and under what circumstances these phagocytes fuse is incompletely understood. Culturing monocytes with IFN-γ *in vitro* reliably results in multinucleation, emphasizing the role of Th1 cells in the formation of giant cells (68). Th1 cells are critical drivers in the M1 differentiation of macrophages and M1 cells have been implicated in the formation and/or maintenance of granulomas (69). One study has suggested that DC fusion takes place under the influence of autocrine IL-17 and exogenous IFN-γ (56), proposing that the concerted action of several cytokines is necessary to promote the optimal granuloma function. In sarcoidosis, believed to be a Th1-dependent disease, granulomas are more inflammatory than suture granulomas or fungal granulomas, based on significantly higher production of IL-6, CCL2, IFN-γ, and Nox2 (70).

The role of TNFα in the formation of granulomas is debated. In tuberculosis, some studies have shown that TNFα induces the formation of granulomas (71) and that TNFα deficient mice have a more severe *M. tuberculosis* infection, possibly due to deficient granuloma formation and thus inability to contain the infection (72). Administering anti-TNFα medication in humans, however, does not appear to suppress granulomatous inflammation (73) or cause disassembly of existing granulomas more effectively than corticosteroids alone (74).

Increased numbers of IL-17 positive cells have been found in granulomas in patients with sarcoidosis (75), suggesting that Th17 cells may participate in the assembly of sterile granulomas. Alternatively, the cytokine milieu of granulomas may provide ideal conditions to polarize peripheral T cells toward the Th17 lineage.

Over time, granulomas can become necrotic or fibrotic. It is unknown which factors regulate the progression of the granulomatous reaction. Differences in the architecture, in the tissue distribution, and in the persistence of granulomas strongly support the notion that granuloma formation is distinct in different disease states. As a common rule, granulomatous infiltrates reflect an intense immune response, associated with marked inflammation and potential of tissue damage.

## Efferocytosis

Besides being a site of concentrated cytokine production, granulomas have a critical function in debris removal, including the removal of apoptotic cells; a process named efferocytosis (76). It is conceivable that the inefficiency of efferocytosis could lead to the persistence of the granulomatous reaction and this could be particularly important in disease states characterized by sterile granulomas. The notion of ineffective phagocytosis in granulomatous disease was suggested in the past (77). Impaired efferocytosis has been described in chronic granulomatous disease, typically associated with a hyperinflammatory state (78). The deficiency of efferocytosis has been related to a defect in phosphatidylserine (PS) signaling and has been reversed by treating macrophages with IL-4, essentially skewing them toward M2.

## Granuloma Formation and Hypervitaminosis D

Patients with granulomatous disease can present with hypercalcemia, considered a result of hypervitaminosis D. This excess vitamin D originates from the granulomas, most likely from the macrophages within. In sarcoidosis, macrophages can produce vitamin D and this production is increased upon stimulation with IFN-γ (79). In line with these findings, a TLR-mediated microbicidal pathway has been reported to upregulate the vitamin D receptor (VDR). The enzyme converting 25-hydroxyvitamin D into the active form of vitamin D, 1,25-hydroxyvitamin D, known as CYP27b1, is also upregulated upon activation of the above mentioned microbicidal pathway (80). In tuberculosis, IFN-γ can increase the production of autocrine IL-15, upregulating CYP27b1 and expression of the VDR. Upon inhibiting the VDR on monocytes, a reduction in autophagy as measured by LC3-positive vesicles was noted. The authors, therefore, suggest that enhanced production of vitamin D and the upregulation of the VDR in monocytes after IFN-γ stimulation result in maturation of the phagosome (81). It is unknown whether the vitamin D pathway is impaired or hyperactive in patients with sterile granulomatous disease.

Interestingly, in GPA and in sarcoidosis variations of disease prevalence patterns according to hours of sunlight per year (and thus dermal vitamin D production) have been discussed. Also, less exposure to sunlight and/or low levels of vitamin D increases the risk for developing GPA or a disease relapse (82, 83).

## Giant-Cell Arteritis

**Figure 2:**
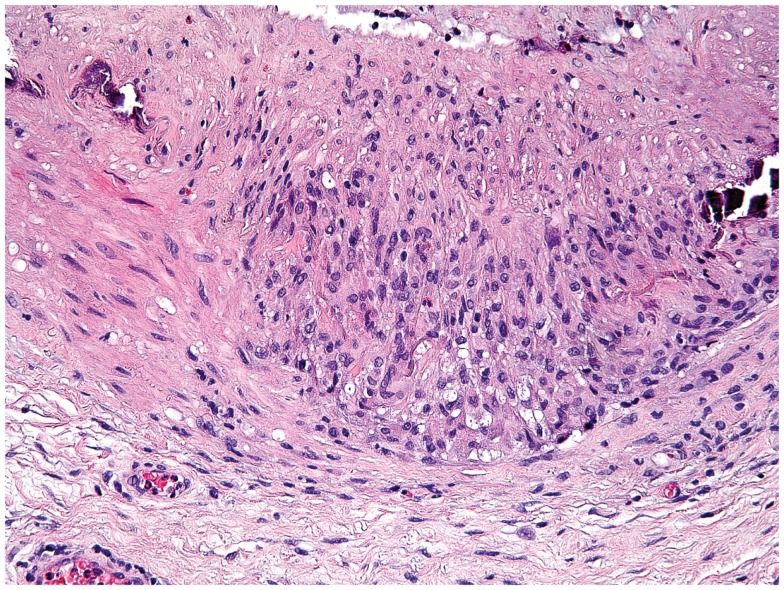
**Histological section showing a granulomatous infiltrate in the temporal artery of a 77-year-old patient with giant-cell arteritis**. The granulomatous reaction is localized at the media-intima border and includes fragments of the lamina elastica externa. Lymphocytes are surrounding highly activated macrophages and giant cells (hematoxylin and eosin staining).

Giant-cell arteritis is a medium- to large-vessel vasculitis almost exclusively diagnosed in patients older than 50 years (84). In brief, presenting symptoms are such of an intense acute phase response; e.g., fever, malaise, weight loss, and laboratory abnormalities of systemic inflammation combined with manifestations of tissue ischemia. It mainly affects extracranial and upper extremity branches of the aorta (e.g., temporal arteries, axillary arteries) and the aorta itself (85). The gold standard for diagnosis remains the biopsy of the temporal artery (86). Typical histopathological findings are granulomatous lesions and/or lymphomonocytic infiltrations in the vessel-wall layers, often containing multinucleated giant cells (87) (Figure 2). In contrast to tuberculous granulomas and granulomas found in Crohn’s disease [the latter assumed to result from a host–commensal bacteria homeostasis gone awry (88, 89)], granulomatous lesions in GCA have not been connected to an infectious agent (90). Reported associations between GCA and pre-existing infection with parvovirus B19, *Chlamydia trachomatis* and *Mycoplasma pneumonia* have raised the possibility of an infectious trigger, but subsequent studies could not always confirm these associations (91). Nevertheless, there is strong evidence for antigen-driven chronic T cell responses (92), particularly Th1 and Th17 responses (93). The induction of Th1 cells in GCA may originate from excessive IL-12 production, which has been found increased in GCA lesions during active disease and during remission (94). The source of IL-12 in GCA remains obscure, but DC have recently been described to produce high levels of IL-12 (95).

Dendritic cells and macrophages are obligatory components of the granulomatous infiltrates in the wall of GCA-affected arteries (96). DC are thought to act as gate-keepers of the vasculitis by inducing T cell activation, and display a phenotype of strong immune-stimulatory APC (92). It has been demonstrated that vessel-wall residing DC are specifically activated via TLR4 or TLR5 (97), enabling them to activate p38 MAPK, and activate downstream TNFα and IL-1β (98).

Accordingly, serum IL-6 and IL-1β levels have been found strongly increased in patients with active GCA (74, 99) and associated with disease activity (100, 101). While the precise cellular source of the excess IL-6 and IL-1β has not been determined, highly activated macrophages and DC in the vasculitic lesions emerge as the likely producers (102). There is evidence that the granulomatous infiltrates in GCA contribute to IL-6 secretion (103), together with production of TGF-β, another well-known product of activated macrophages (104). IL-6 levels in GCA patients decrease upon treatment with corticosteroids, but remain higher in some patients when compared to healthy controls, indicating a more chronic course of disease (99).

Recently, IL-33 was reported to be increased in vessel-wall lesions of patients with GCA (105). IL-33 is secreted by endothelial cells when under stress or becoming apoptotic. This stress possibly results from high levels of circulating IL-6. Importantly, IL-33 has been found to favor M2 polarization of macrophages, implicating this macrophage subset in the pathogenesis of GCA (106).

Giant-cell arteritis is considered an antigen-dependent disease in which Th1 cytokines dominate and Th2 cytokines are generally absent. Moreover, during early and untreated disease, Th1 and Th17 cells co-exist in the vasculitic lesions (93) (Figure 2). The granulomatous inflammation may present the platform that allows differentiation of entering naïve T cells toward Th1 and Th17 under the influence of macrophage and DC products (Table 1). By seeding Th1 and Th17 cells into the periphery, granulomas could have a major impact on the composition of the overall immune system. Since differentiated memory cells are long-lived cells, even a temporary granulomatous reaction could permanently remodel the immune system and have long-term implications for the host. During corticosteroid treatment, Th17 cells decrease, but Th1 cells appear to persist (93). In line with these findings, increased serum levels of IL-12, IL-17, IL-21, IL-23, IL-27, IL-32, and IFN-γ have been observed in patients with active GCA (74, 107). Granulomas may represent an important source of IL-17, with elevated protein levels and mRNA observed within the granulomatous vessel-wall infiltrates (108, 109). It is important to consider that the overall frequencies of Th17 cells are low, outnumbered by Th1 cells by a factor of 10 or higher. This may be particularly relevant during the chronic phase of GCA, when persistent inflammation relies on Th1 cells and their major product, IFN-γ. Other Th1 products produced in the inflamed vessel wall include IL-27 and IL-32. Both have been reported to induce M1 cells (107).

**Figure 3:**
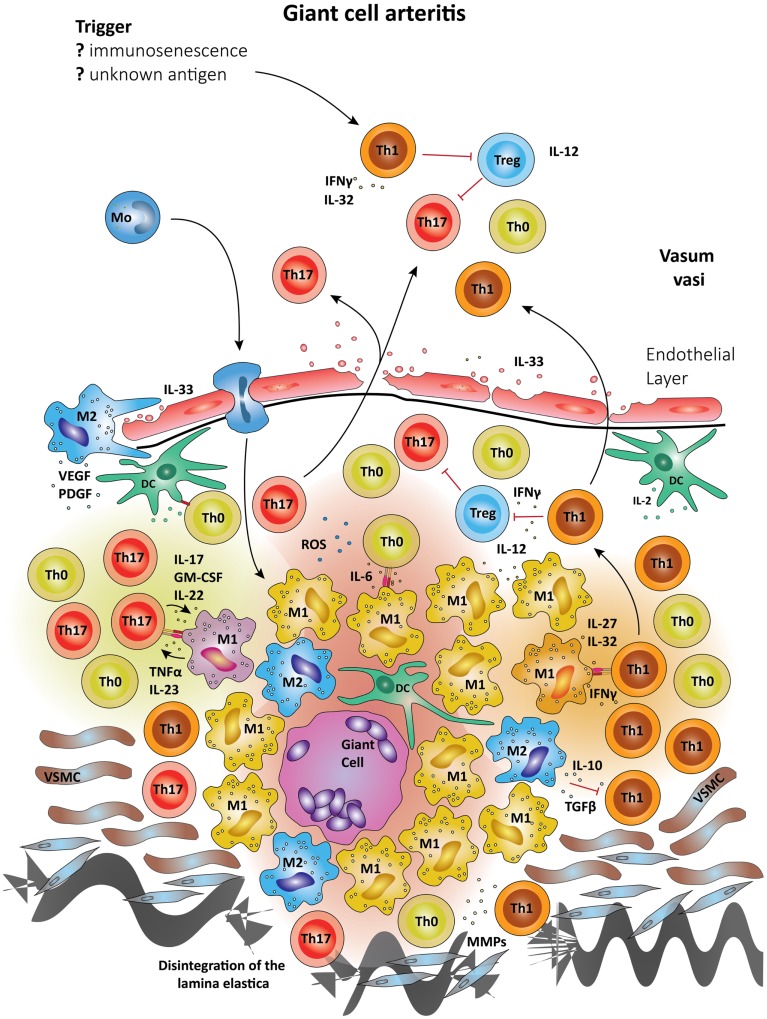
**Schematic summary of the pathogenic pathways implicated in granulomatous lesions in giant-cell arteritis**. The upper panel represents pathogenic events in GCA. All immune recognition events and tissue damage occur within the vessel wall, not the perivascular tissue. Wall-resident DC, so-called vasDC, coordinate the recruitment and the differentiation of macrophages and T cells. IL-12 is instrumental in biasing T cells toward the Th1 lineage; IL-6, TGF-β, and IL-23 provide signals for Th17 differentiation. Dependent on their positioning in the vessel-wall monocytes commit to distinct functional profiles; e.g., metalloproteinases production, release of ROS, secretion of cytokines. Treg are underrepresented, partially due to inhibitory effects from IFN-γ. The lower panel shows the typical localization of granulomatous infiltrates on the adventitia side of the vessel wall. Monocytes (Mo) enter these lesions via the vasa vasorum and mature in the lesions under the influence of specific microenvironments. Different M1 cells are presumed to influence Th0 cells into Th1 and Th17 cells via cell–cell contact and the secretion of cytokines. Activated macrophages and DC may fuse and form multinucleate giant cells, a hallmark of GCA. The granulomatous infiltrate is a highly inflammatory microenvironment, which promotes the differentiation of Th0 to Th1 and Th17, which are then seeded into the circulation. Due to their localization, granulomatous infiltrates in GCA influence vascular smooth muscle cells (VSMCs) and myofibroblasts. The latter expand in number, migrate and result in concentric media hypertrophy. Also, the external lamina elastica disintegrates as a result of damage from ROS and matrix metalloproteases (MMPs). Although M1 are assumed the most frequent in granulomatous infiltrates, M2 cells have been proposed as a counter-mechanism and implicated in supporting tissue repair. Vascular endothelial growth factor (VEGF) and platelet-derived growth factor (PDGF) have been described in the vasculitic lesions of GCA and networks of neoangiogenic microvessels typically accompany the process of intimal hyperplasia (167, 168).

Since the differentiation of T cells into distinct lineages results from the exposure of non-committed T cells to antigen plus polarizing cytokine environment, it is highly likely that macrophages and DC residing in the vessel wall ultimately shape vasculitogenic T cell responses (Figure 3). Differentiation of Th17 cells depends on the combined action of IL-6, TGF-β, and IL-23 (110, 111). Recent data have given rise to the concept that Th17 cells are plastic and, dependent on environmental signals, can be redirected to acquire regulatory T cell (Treg) functions (112). There is some evidence that Tregs may be underrepresented in GCA patients, perhaps explaining the inability of affected patients to clear granulomatous lesions (109).

Granulocyte-macrophage colony-stimulating factor (GM-CSF) is distinctly elevated in the serum of patients with GCA. Terrier et al. have reported that GM-CSF is essential for the production of IL-6 and IL-23 by DC; thus indirectly promoting the generation of Th17 cells (113). DC in GM-CSF^−/−^ mice produce lower levels of IL-6, resulting in deficient proliferation of T cells in general and reduced Th17 differentiation. More recently, it has been reported that Th17 cells produce GM-CSF when stimulated with IL-23 and that this production is upregulated by IL-1β (114).

Given the central role of granulomatous infiltrates in GCA, disrupting granuloma formation should be a valuable therapeutic target. Surprisingly, anti-TNF therapy has failed to reduce steroid requirements or prevent disease flares in GCA patients when combined with corticosteroids (115).

With the knowledge of macrophages and DC forming the basis of granuloma formation, inhibiting these cells directly may prove beneficial in treating GCA; bearing in mind, however, the risk of infectious complications associated with a deficient innate immune system. As mentioned earlier, TLR play an important role in the activation of APC in infectious and in sterile inflammatory settings. TLR may, therefore, represent a therapeutic target to treat sterile granulomatous inflammation by inhibiting granuloma formation and reducing tissue damage (116). Other receptors and APC markers are being studied for their suitability in new therapeutic interventions. Targeting CD14 with anti-CD14 antibodies has been attempted in septic models, but not in autoimmune settings (117). In murine sepsis, blocking the innate immune system results in less inflammation, less intense cytokine storms, and a higher survival rate.

## Granulomatosis with Polyangiitis

**Figure 4:**
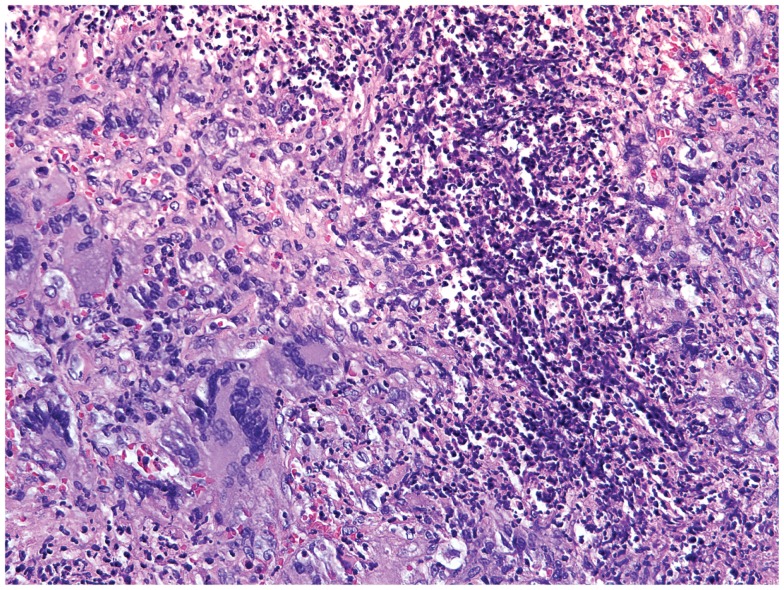
**Histological section of a granulomatous lesion in the lung of a 56-year-old patient with granulomatosis with polyangiitis**. The core of the granuloma consists of multinucleate giant cells, macrophages, and neutrophils and is encircled by a rim of lymphocytes (hematoxylin and eosin staining).

**Figure 5:**
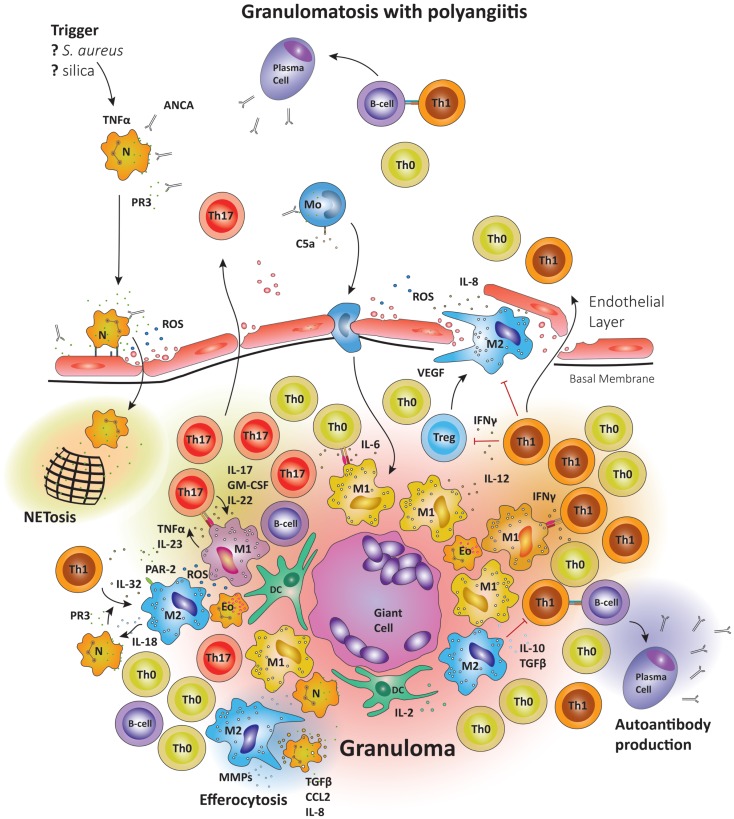
**Schematic summary of the pathogenic pathways implicated in granuloma formation in granulomatosis with polyangiitis**. The upper panel represents the pathogenesis of granulomatosis with polyangiitis (GPA). Upon priming neutrophils bring to their surface high levels of proteinase-3 (PR3) and myeloperoxidase (MPO), the autoantigens recognized by ANCA. The priming process is believed to be mediated by TNFα. TNFα production may be induced by a variety of triggers; e.g., *S. aureus*, silica, etc. Anti-PR3 or anti-MPO antibodies are then able to bind these enzymes on the cell surface, causing neutrophils to degranulate, bind to endothelial cells, enter the perivascular tissue, and release ROS; thereby damaging the vessel wall. In the tissue, neutrophils releases so-called neutrophil extracellular traps (NETs), which are networks of fibers and DNA to trap pathogens. Many of the highly activated neutrophils become apoptotic and are then phagocytosed by resident M2 macrophages, a process that has been named efferocytosis and that may be deficient in GPA. During efferocytosis, macrophages release TGF-β, IL-8, and CCL2. Monocytes may also be activated by circulating ANCA, enhancing their chemotactic responsiveness and enabling them to participate in granuloma formation. The lower panel represents the formation of a sterile granuloma in extravascular tissue. Monocytes and CD4^+^ cells enter the granuloma following a gradient of chemokines and cytokines; a process sufficient to transform monocytes into macrophages. Commitment to the M1 or M2 lineage is dependent on the specific cytokine environment. Neutrophils and eosinophils are commonly found in granulomas in GPA. Also, B cells have been reported in the surroundings of granulomas, where they may undergo further maturation. Multinucleate giant cells are present within the granulomas, resulting from the fusion of either macrophages or dendritic cells. The organized arrangement of the granuloma provides an ideal platform for macrophage–T cell interaction. CD4^+^ cells coming into contact with IL-6 and TGF-β producing M1 cells are skewed toward the Th17 lineage. M1 also secrete IL-23; sustaining the Th17 population. In turn, Th17 cells can secrete granulocyte and GM-CSF in addition to IL-17 and IL-22, thus stabilizing M1 differentiation. However, M2 cells are equally represented in granulomas and secrete IL-10 and TGF-β. M2 cells are a source of vascular endothelial growth factor (VEGF) and support the outgrowth of microvessels, critically important as the granuloma grows in size.

Small-vessel vasculitides associated with the production of autoantibodies reactive to proteinase-3 (PR3) or myeloperoxidase (MPO) are collectively called anti-neutrophil cytoplasmic antibodies (ANCA)-associated vasculitides (AAV). The group of AAV encompasses GPA, microscopic polyangiitis, and eosinophilic GPA (118). The pathogenesis of these vasculitides is incompletely understood, but great progress has been made in diagnosis and therapy of these autoimmune diseases. Yearly incidence rates are estimated at 20 cases per million individuals (119). A high mortality and (co)morbidity unfortunately persists despite improvement of therapy and knowledge of the disease process (120–122). In GPA, granulomas are typically found in the ear, nose, and throat region, in the lungs, periglomerularly in the kidneys, and more rarely in other organs (123) (Figure 4). The autoantigens recognized by the autoantibodies in AAV are intracellular enzymes produced by neutrophils and monocytes. In both cell types, PR3 and MPO are expressed on the cell membrane in low levels in healthy controls and in aberrantly high levels in AAV patients, especially during active disease (124, 125). Over 75% of GPA patients are PR3-ANCA positive with the remainder being either MPO-ANCA positive or ANCA-negative, especially in patients of Caucasian descent (126). Interestingly, when monocytes differentiate into macrophages they lose expression of PR3 and MPO on their cell membrane. Thus, monocytes can be activated by ANCA (127), whereas mature macrophages cannot (128), placing antibody-dependent disease mechanisms early into the pathogenic immune response (Figure 5).

The neutrophil, playing a central role in the pathogenesis of GPA, degranulates once ANCA bind to surface PR3 or MPO (129). Neutrophils then become apoptotic and are cleared by macrophages. While dying, neutrophils excrete so-called neutrophil extracellular traps (NETs), physiologically meant to “trap” pathogens in a network of decondensed chromatin, MPO, PR3, and other enzymes, but causing damage in the case of GPA by releasing more autoantigen into the granulomatous lesion (130). The clearance of apoptotic neutrophils by macrophages has been termed efferocytosis (see above) and is associated with secretion of a variety of cytokines and chemokines, including TNFα and prostaglandin E_2_ (PGE_2_) (131). Strongly activated macrophages have been localized within and around glomeruli affected by pauci-immune necrotizing crescentic glomerulonephritis, the pathognomonic lesion of AAV (132). These macrophages have been found to produce IL-18, thus attracting more neutrophils to the granulomatous inflammation (133). Such, activated macrophages express high levels of HLA class II molecules, enabling them to act as highly efficient APC (132). Of the leukocytes infiltrating the glomeruli and the tubulointerstitium, macrophages are the most prevalent, followed by granulocytes (134), differentiating GPA from GCA, in which granulocytes essentially do not participate. In renal biopsies from patients with active GPA, DC, and macrophages are localized in glomeruli and in periglomerular infiltrates, whereas DC and macrophages are absent from normal renal tissue. DC and T cells appear to interact within the periglomerular infiltrates (135), consistent with antigen recognition events orchestrating the tissue-damaging immune responses.

Moderately elevated levels of serum IL-1β and IL-6 have been reported in GPA patients during active disease, in line with persistent activation of macrophages in the tissue lesions (136–138). IL-1β and IL-6 are produced by M1 (138), but also by damaged endothelial cells (139). Serum levels of IL-1β, TNFα, and sIL-2R have been correlated with the presence of corresponding mRNA in tissue lesions, suggesting that mononuclear cells in vasculitic lesions are the origin of these proinflammatory mediators (140). In a mouse model of small-vessel vasculitis, IL-1β produced by monocytes has been associated with glomerulonephritis and blocking the IL-1β receptor with anakinra has resulted in a decrease of cellular crescents and hematuria (141).

The enzyme PR3 may play a central role in granuloma formation since it was found to be capable of cleaving IL-32, thereby enhancing its activity. IL-32 is produced by Th1 cells and its active form results in macrophage activation, leading to TNFα and IL-8 production (142). In addition, PR3 can cleave the protease-activated receptor 2 located on the cell surface of macrophages, leading to downstream inflammation, particularly IL-18, CXCL2, and IL-8 (142, 143). IL-18 is a known neutrophil attractant (Figure 5).

In search for the mechanisms through which autoantibodies mediate pathology in GPA, PR3-ANCA have been shown to induce upregulation of TLR2, 3, 4, 7, and 9, as well as NOD-1 and NOD-2 (144). Nucleotide-binding oligomerization domain-containing protein 2 (NOD-2) is an intracellular pattern-recognition molecule enabling macrophages to recognize bacterial molecules that contain muramyl dipeptide (MDP). In contrast to Crohn’s disease, where mutations in *NOD2* have been implicated as disease risk factors (145), no such mutations were found in GPA patients (146). Interestingly, however, another study showed an association between mutations in the *TLR9* gene and PR3-ANCA positivity as opposed to MPO-ANCA positivity (147). In mice, ligands for TLR2 and TLR9 have both been implicated in kicking off autoimmunity. Notably, ligands for TLR2 have led to an expansion of Th17 cells, whereas ligands for TLR9 preferentially facilitate the expansion of Th1 cells (148). A possible role of TLR2 ligands in the pathogenesis of GPA is supportive of an involvement of *Staphylococcus aureus* in the development (83) as well as the risk for relapse in GPA (149), as *S. aureus* is a known ligand for TLR2 (150). In support of this notion, monocytes from patients with GPA have been found to express higher levels of TLR2 on the cell surface (151). Genetic factors are likely to be associated with the process of granuloma formation: these factors remain difficult to establish. One study found an association between the PTPN22 R620W polymorphism and the presence of granulomatous lesions (especially in the ENT region) specifically in patients with GPA, as opposed to patients with MPA or EGPA (152).

A macrophage product previously associated with granuloma formation in tuberculosis and silicosis is osteopontin (153). Interestingly, osteopontin is elevated in patients with active GPA (154) and has been detected in crescentic lesions in glomeruli (155). Osteopontin production has been associated with macrophage activation and it has been proposed that this monokine functions as a monocyte chemoattractant, securing the influx of fresh monocytes into the granulomatous lesions. Importantly, osteopontin synthesis is stimulated by active vitamin D, the latter, as mentioned above, a signifying product of active granulomas (156). There is evidence that osteopontin may play a role in TLR4-mediated IL-10 production in T cells, which suppress macrophages (157), delineating a negative feedback loop via which T cells can minimize granuloma-associated tissue damage.

Considering the destructive consequences of granuloma formation in GPA, it would be advantageous to treat GPA patients with therapies inhibiting this process. Studies on the use of anti-TNFα treatment in GPA patients have suggested that it may have a place in induction therapy (158), but patients remained at a higher risk for relapse (159). Depletion of B cells by anti-CD20 antibodies provides effective immunosuppression in patients with GPA (160). Whether this therapeutic approach functions by depressing autoantibody-dependent mechanisms or whether B cells provide other disease relevant functions, such as cytokine production and antigen presentation, remains speculative. It is conceivable that B cells are critically involved in toning the immune system and that their depletion impairs innate as well as adaptive immunity. Directly targeting macrophages may hold promise in immunosuppressing patients with GPA, although loss of macrophage function may further weaken their ability to mount protective immunity, especially against microbial pathogens.

## Future Directions

It is currently unknown whether macrophages trapped in granulomas can be easily assigned to a functional lineage, e.g., M1 and M2, or whether residence in a granuloma directs macrophages into a separate differentiation program. Circulating cytokines in granulomatous disease favors the concept that the majority of macrophages may be M1. However, M2 have been localized in granulomatous lesions. This may simply be a negative feedback mechanism to temper inflammation in order to prevent excessive tissue damage. Indeed, one study found both M1 and M2 in the vessel lesions in GCA patients (105). A distinguishing feature of granulomas is the high cell turnover, giving rise to the need to clear apoptotic short-lived cells, such as neutrophils and macrophages. Effective removal of apoptotic bodies relies on the process of efferocytosis, again placing macrophages at a center stage. The current paradigm suggests that mainly M2 are responsible for effective efferocytosis. The intactness of these mechanisms in granulomatous vasculitis is unknown, but it could be hypothesized that a major defect lies in the inability of the patients to turn down M1 macrophage activation and bring to bear M2 macrophages.

Interestingly, GCA and GPA are distinct diseases affecting different sizes of blood vessels, but they are both characterized by granulomatous lesions. The granulomatous infiltrates in GCA are predominantly located in the vessel wall, where monocytes arrive via the vasa vasorum (87). In contrast, the granulomatous lesions in GPA are more often extravascular and, in the case of renal involvement, periglomerular (161). The classic granuloma with palisading as described by Godman and Churg in 1954 (162), can be found in GPA but not in GCA. The overall architecture of granulomas, characterized by an inner core of macrophages and DC surrounded by T cells, however, is present in both vasculitides. Importantly, the cellular composition of GCA- and GPA-associated granulomas seems to be different. Due to the intramural localization of the granulomatous lesion in GCA, vascular smooth muscle cells (VSMCs) and myofibroblasts are in intimate relationship to the granuloma-forming immune cells. Neutrophils are found exclusively in granulomas of GPA patients, as well as eosinophils. Also, B cells are absent from lesions in GCA (163) but they can be found in granulomas of GPA patients (164), where they may be able to mature and contribute to B cell dependent pathology. It has been proposed that the granuloma in GPA may participate in autoantibody production (165).

Granulomatous vasculitides reflect abnormalities in both, the innate and adaptive arm of the immune system. Activation products of innate cells, in particular cytokines, have attracted much attention as potential biomarkers of disease and effort has been invested to test whether they can help in quantifying disease burden. Similarities in the abnormal immune reactions of distinct vasculitides make it unlikely that a single cytokine will emerge as a disease-specific biomarker. However, cocktails of cytokines may have value in assessing how active the disease is in individual patients. Quantifying adaptive immunity beyond antibody formation has been challenging. In AAV, autoantibodies against PR3 and MPO have served an important diagnostic role, as they can help in rapidly reducing differential diagnosis in acutely sick patients. There is currently insufficient evidence that the titer of these autoantibodies is a good marker of disease activity. During the chronic course of AAV, autoantibody titers have limited use in helping make therapeutic decisions (166).

The possibility remains that granulomas will guide the search for the disease inducing antigens; as such antigens should be enriched in these tissue sites. Understanding the mechanisms of granuloma formation and the role of these lymphoid microstructures in perpetuating pathology could greatly enhance the spectrum of therapeutic targets. In all inflammatory vasculopathies, corticosteroids remain a cornerstone of therapy. Their therapeutic benefit may mainly result from their ability to suppress macrophage function. Temporary suppression of macrophage function, while effective in reducing acute phase responses, has little impact on the long-lived cells of the adaptive immune system, and thus fails to induce durable remission.

There remain considerable challenges in optimizing the management of patients with inflammatory blood vessel disease, despite enormous progress in deciphering processes of innate and adaptive immunity. The initial triggers derailing host protective immunity are undetermined. Given the importance of granuloma formation in protective and pathogenic immunity, speculations about infectious agents setting off vasculitis have held steady over decades. Hopes for the identification of such a disease inducer have not been met with success. Clustering of risk in geographic regions and populations have nurtured the belief that genetic risk factors are important, but could equally well support the role of environmental determinants. Most of the vasculitides are HLA associated diseases, providing further support for a critical contribution of antigen recognition and adaptive immunity in pathogenesis. Granulomas remain fascinating structures that bring together innate and adaptive immune cells and may ultimately hold the key to understanding why the power of immune protection is misused to harm the host.

## Conflict of Interest Statement

The authors declare that the research was conducted in the absence of any commercial or financial relationships that could be construed as a potential conflict of interest.
